# Vitamin C Transporters in Cancer: Current Understanding and Gaps in Knowledge

**DOI:** 10.3389/fonc.2017.00074

**Published:** 2017-04-24

**Authors:** Christina Wohlrab, Elisabeth Phillips, Gabi U. Dachs

**Affiliations:** ^1^Mackenzie Cancer Research Group, Department of Pathology, University of Otago, Christchurch, New Zealand

**Keywords:** ascorbate, sodium-dependent vitamin C transporter 1, sodium-dependent vitamin C transporter 2, tumor, expression

## Abstract

Sufficient uptake and whole body distribution of vitamin C (ascorbate) is essential for many biochemical processes, including some that are vital for tumor growth and spread. Uptake of ascorbate into cancer cells is modulated by availability, tumor blood flow, tissue diffusion parameters, and ascorbate transport proteins. Uptake into cells is mediated by two families of transport proteins, namely, the solute carrier gene family 23, consisting of sodium-dependent vitamin C transporters (SVCTs) 1 and 2, and the SLC2 family of glucose transporters (GLUTs). GLUTs transport the oxidized form of the vitamin, dehydroascorbate (DHA), which is present at negligible to low physiological levels. SVCT1 and 2 are capable of accumulating ascorbate against a concentration gradient from micromolar concentrations outside to millimolar levels inside of cells. Investigating the expression and regulation of SVCTs in cancer has only recently started to be included in studies focused on the role of ascorbate in tumor formation, progression, and response to therapy. This review gives an overview of the current, limited knowledge of ascorbate transport across membranes, as well as tissue distribution, gene expression, and the relevance of SVCTs in cancer. As tumor ascorbate accumulation may play a role in the anticancer activity of high dose ascorbate treatment, further research into ascorbate transport in cancer tissue is vital.

## Ascorbate and Cancer

The role of vitamin C (ascorbate) in cancer risk, progression, and therapy is not resolved (1–3), but cancer patients are frequently ascorbate deficient (4, 5). Ascorbate’s role is likely to involve a combination of several of the following proposed functions: (a) by acting as an electron donor it is a potent antioxidant (6), and has thus been proposed to interfere with some chemotherapy regimes (7); (b) ascorbate has prooxidant properties *via* its ability to reduce redox-active metals (8), and thus is proposed to directly act as a cytotoxin (9); and (c) ascorbate serves as a cofactor for a large family of Fe(II) and 2-oxoglutarate-dependent dioxygenases, which includes the collagen prolyl hydroxylases required for the formation of the tertiary structure of collagen, regulation of hypoxia-inducible transcription (HIF) factors required for tumor angiogenesis, treatment evasion and metastasis, and nucleotide hydroxylases involved in DNA demethylation, which affects global gene expression (10).

In cancer, ascorbate’s function as cofactor for the HIF hydroxylases is currently the most plausible, with several lines of evidence. Studies have shown that (a) in cultured cells, increasing intracellular ascorbate decreased HIF-1 activation (11–13), (b) in ascorbate-dependent mouse models, increasing circulating ascorbate *via* dietary intervention or ascorbate injections reduced tumor growth and hypoxia, and dampened HIF-1 activity (14–16), and (c) in tumor tissue from cancer patients with endometrial and colorectal cancer, increased ascorbate levels were associated with reduced HIF-1 pathway activity (17, 18). Importantly, tumor levels of ascorbate were associated with improved disease-free survival in colorectal cancer patients (18).

## Ascorbate Uptake into Tumors

Uptake of ascorbate into cancer cells is modulated by availability, tumor blood flow, tissue diffusion parameters, and ascorbate transport proteins. Potential limiting factors to ascorbate uptake into solid tumors include (1) tissue characteristics, such as high interstitial fluid pressure and high cell density, (2) suboptimal vascular function, which includes avascular regions, immature and dysfunctional vessels, and varied blood flow, and (3) transporter concentration, location, and function. Data derived from a three-dimensional cellular diffusion model have suggested that ascorbate penetration into a poorly vascularized tumor may require higher than normal plasma concentrations (19). A recent pharmacokinetic study in mice has shown that in response to a single administration of high dose ascorbate, there was a prolonged presence of the vitamin in the tumor compared to plasma and liver, where ascorbate levels reduced rapidly (16), indicating that tumor accumulation may be different from normal tissue.

**Figure 1:**
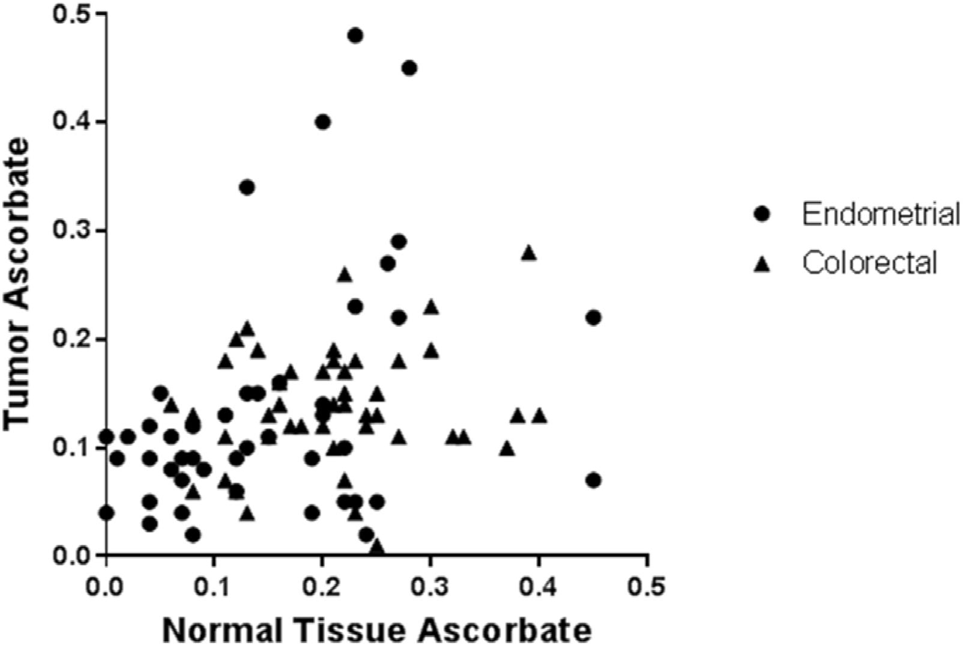
**Ascorbate levels in normal tissue are not associated with ascorbate levels in tumors from endometrial and colorectal cancer patients**. Tissue samples from patients with endometrial (*n* = 50) and colorectal cancer (*n* = 50) were processed, and ascorbate levels (nanomoles per microgram DNA) were measured using HPLC-EC [data from Ref. (17, 18)]. From each patient, samples were obtained from tumor and adjacent normal tissue. There was no association between ascorbate content in tumor vs. normal tissue in individual patients with endometrial or colorectal cancer (Pearson correlation: *R*^2^ = 0.001, *p* = 0.81, and *R*^2^ = 0.022, *p* = 0.30, respectively). Hence, whole body ascorbate status may not predict tumor ascorbate status.

In patients with colorectal or endometrial cancer, where ascorbate levels of both tumor and uninvolved adjacent normal tissue were measured, no association between ascorbate levels in tumor tissue and matched normal tissue was apparent (Figure 1) (17, 18), indicating differences in tumor uptake. Transporter status of these clinical samples was not reported.

## Ascorbate Transport and Elimination

Maintenance of whole body ascorbate levels and distribution to different compartments is mediated by two families of transport proteins (20). While ascorbate is taken up into cells *via* sodium-dependent vitamin C transporters (SVCTs), its oxidized form, dehydroascorbate (DHA), is accumulated through facilitated diffusion *via* glucose transporters (GLUTs) (21, 22). A small proportion of ascorbate and DHA can also pass the plasma membrane *via* passive diffusion (23, 24).

Increased formation of DHA in the tumor microenvironment through oxidative stress (25) or *via* parenteral administration of high doses of DHA (26) has been proposed to enable the accumulation of ascorbate in cancer cells *via* GLUTs, but this has not been measured or proven. GLUT1 overexpression is associated with *KRAS* or *BRAF* mutations in colorectal tumors, thus potentially increasing DHA uptake in this important subset of patients (25). Here, KRAS-mutant colorectal cancer cells in culture preferentially took up DHA rather than ascorbate and were selectively sensitive to and killed by DHA (25). Yet, in a different study using a range of KRAS-mutant colorectal cell lines, ascorbate transport, and sensitivity was reliant on SVCT2, not GLUTs (27). Due to very low or undetectable physiological DHA concentrations in plasma (<10% of total ascorbate) (28, 29), its uptake is likely to be of minor importance *in vivo*, and DHA transport is therefore not discussed further in this review.

How ascorbate exits cells is not fully understood. Different mechanisms have been suggested, including volume-sensitive and Ca^2+^-dependent anion channels, gap-junction hemi-channels, exocytosis of secretory vesicles containing ascorbate, and homo- and hetero-exchange systems at the plasma membrane (30–33). Ascorbate efflux from, as opposed to uptake into cancer cells, and tumors as a whole, has not been studied.

Sodium-dependent vitamin C transporters are members of the solute carrier gene family 23 (*SLC23*), and each protein is comprised of 12 transmembrane domains (34). Currently, three isoforms have been identified, SVCT1 and 2 transport ascorbate, and the orphan receptor SVCT3 has still unknown function (35). SVCT1 and SVCT2 exert the cotransport of sodium and ascorbate in a ratio of 2:1 down an electrochemical sodium gradient, which is maintained by K/Na^+^ exchange mechanisms (36). This transport is sensitive to changes in temperature and pH with an optimum at pH 7.5 (37, 38). SVCT2 also relies on Ca^2+^ and Mg^2+^ for its activity (36). Expression of the different SVCT transport proteins is tissue and cell type specific and is controlled by transcriptional regulation of *SLC23* genes (29, 35) and posttranslational regulation of the transporters (39). However, the exact regulation mechanisms of ascorbate transport proteins are still not fully understood.

The characteristics of SVCT1 and SVCT2, and their known relevance to cancer, are described in the next sections.

## Sodium-Dependent Vitamin C Transporter 1

### Distribution in Normal and Tumor Tissues

Sodium-dependent vitamin C transporter 1 (SLC23A1) is expressed in the epithelial tissue of kidney, intestine, liver, lung, and skin. In the kidney, SVCT1 is situated in the brush-border membrane of the proximal tubule where it mediates reuptake of ascorbate, thereby playing a major role in maintenance of whole body ascorbate levels (40). SVCT1 transports ascorbate with low affinity with a *K*_m_ in the range of 65–237 µM, which makes it capable of high capacity uptake of ascorbate from the diet (36, 41). Only one study has measured SVCT1 protein and ascorbate levels in mouse tumors and reported low SVCT1 levels with little variation following ascorbate administration (16). Tissue distribution of SVCT1 in cancer patients has not been described.

### *SLC23A1* Polymorphisms and Cancer Risk

The gene (*SLC23A1*) is located on chromosome 5 and contains binding sites for the hepatocyte nuclear factor 1 transcription factor in its promoter region (42). In humans, no loss of *SLC23A1* has been described to date. However, different single nucleotide polymorphisms (SNPs) in the coding region have been identified impairing ascorbate transport and reducing plasma levels (29, 41, 43). Studies focusing on the association of *SLC23A1* variants and cancer risk gave conflicting results (29, 43–45). SNPs have been linked to increased risk of follicular lymphoma (rs6596473 G>C) (44), with no influence on gastric cancer (43) or advanced colorectal adenoma (45). However, plasma ascorbate levels were not measured in these reports, and further investigations are needed, to evaluate the link of *SLC23A1* polymorphisms with disease risk.

### Control of Gene Expression

Gene and protein expression studies of SVCT1 in cancer have not been widely reported. *Svct1* mRNA levels were upregulated by bile acids in rat hepatoma cells (46), whereas glutathione depletion resulted in decreased *Svct1* mRNA, protein levels and ascorbate transport (47). Human hepatoma cells, which unlike rat are incapable of synthesizing ascorbate, were unaffected by glutathione depletion (47). Inconsistent results were reported for colorectal cancer cells. In human colon carcinoma cells, high concentrations of ascorbate downregulated *SVCT1* expression *in vitro* (48), while no difference in SVCT1 protein expression was observed in human colon adenocarcinoma samples compared to normal colon mucosa (49).

### Posttranslational Modification

Ascorbate transport is not only regulated at the level of gene expression of *SLC23A1* (42) but also *via* posttranscriptional regulation, and glycosylation and phosphorylation regulate SVCT1 activity (39). Translocation of SVCT1 carriers from the cytosol to the cell membrane was detected in human keratinocytes upon UVB irradiation, without changes in mRNA expression, indicating that cellular localization of SVCTs may be an important determinant of the ascorbate uptake rate (50). Yet, studies in cancer cells have not been conducted.

## Sodium-Dependent Vitamin C Transporter 2

### Distribution in Normal and Tumor Tissues

Sodium-dependent vitamin C transporter 2 is expressed in almost every tissue and cell in the body (29). It has been characterized as a low capacity, high affinity transporter with a transport *K*_m_ of ~20 μM and can thus take up lower concentrations of ascorbate than SVCT1 (36). The SVCT2 transporter is highly expressed in the brain where it is essential for maintaining the high ascorbate levels needed for brain function and development (51, 52). In human bronchial epithelium, SVCT2 protein expression inversely correlated with ascorbate concentration in the respiratory tract lining fluid (53). SVCT2 protein was readily detected in Lewis lung tumors grown in ascorbate-dependent mice, and SVCT2 protein levels varied over time following a single high dose ascorbate injection, but their association with tumor ascorbate levels was complex (16). No other studies have measured SVCT2 in tumor tissue.

### *SLC23A2* Polymorphisms and Cancer Risk

Sodium-dependent vitamin C transporter 2 is encoded by the *SLC23A2* gene located on chromosome 20. A short isoform of SVCT2, naturally occurring in humans through alternative splicing, is unable to transport ascorbate (54). It has been shown to negatively regulate the function of the full length transporter by changing its affinity constant *via* hetero-oligomerization in human embryonic kidney 239T cells and mouse neuronal cells (54). This isoform also had the ability to partially inhibit SVCT1 (54, 55).

Several studies have focused on *SLC23A2* gene polymorphisms related to ascorbate levels and disease risks. Two SNPs located in the intron region of *SLC23A2* (rs6133175, rs1776948) were associated with risk of chronic lymphocytic leukemia (CLL) in a case–control study (*n* = 1,691) (56). However, no relationship between CLL risk and dietary ascorbate intake, as determined *via* questionnaires, was detected, and ascorbate was not measured (56). Another SNP (rs12479919) was inversely correlated with gastric cancer risk in a Polish study cohort (*n* = 693) (57). Similarly, in a European study (*n* = 365 cases, 1,284 controls), *SLC23A2* SNPs (rs6053005, rs6133175) were predictive of plasma ascorbate levels, and haplotype variants were associated with risk of gastric cancer (43). In Japanese patients with esophageal squamous cell carcinoma (*n* = 49), two SNPs (rs268116, rs13037458) tended to associate with clinical response and long-term survival after 5-fluorouracil (5-FU)/cisplatin-based chemoradiotherapy, and two SNPs (rs4987219, rs1110277) correlated with chemotherapy-induced toxicity (58). Although several SLC23A family members have been reported to transport nucleobases, such as 5-FU, human SVCT1 and 2 reportedly do not possess this activity (59). Interestingly, *SVCT2* mRNA levels also correlated with sensitivity to 5-FU in human esophageal cancer cell lines (60). Analysis of another polymorphism (rs4987219) revealed no association with cancer risk in squamous cell carcinoma of the head and neck in a Brazilian study (*n* = 165, 230 controls) (61). In neither study, ascorbate was measured.

### Control of Gene Expression

Similar to *Svct1*, mRNA expression of *Svct2* was upregulated by bile acids in rat hepatoma cells *in vitro* (46), while glutathione depleted cells had decreased *Svct2* mRNA and protein levels (47). In human hepatoma cells, however, neither bile acids nor glutathione depletion had an effect on *SVCT2* expression (46, 47). In human breast cancer cells, *SVCT2* mRNA levels differed significantly between cell lines (62).

### Subcellular Localization of SVCT2

The localization of SVCT2 within the cell may determine its transport activity, although data are conflicting. SVCT2 protein can be retained within intracellular compartments or transport vesicles and may have different kinetic properties depending on its localization (63), but data in cancer cells are limited. In neurons in culture, SVCT2 translocated to the plasma membrane upon increased extracellular ascorbate concentration (64). In a murine model of Huntington’s disease, impaired membrane translocation of SVCT2 led to insufficient accumulation of ascorbate in neurons (64).

Mitochondrial SVCT2 has been proposed to function as a low-affinity transporter due to differences in intracellular sodium and potassium concentrations (65). Lower sodium and higher potassium concentration inside embryonic kidney cells increased the transport *K*_m_ from 20 µM to over 600 µM in mitochondria, and this was proposed to make transport more responsive to variations in intracellular ascorbate levels, and enabling transport into intracellular organelles (65). A transporter with a *K*_m_ of 20 µM or less, as it is the case for SVCT2 in the plasma membrane, would function at maximal velocity at 200 µM ascorbate and not be able to respond to millimolar intracellular concentrations (65). In contrast, in U937 human myeloid leukemia cells, SVCT2 had similar kinetic characteristics at both locations (66). It was thus concluded that the transporter might function with different affinities in different cell types.

### SVCT2 Activity

Data in mice showed increased radioactive ascorbate uptake in adrenal glands, compared to adrenocortical or adrenal medulla tumors, and adrenal uptake was sensitive to the ascorbate transport blocking agent sulfinpyrazone (67). In a mouse neuroblastoma cell line, SVCT2 was found to have two different transport kinetics (*K*_m_ of 13 and 105 µM), and transport could be inhibited with flavonoids (68). Expression of SVCT2 in human neuroblastoma tissue was confirmed by immunofluorescence (68) but activity is unknown. SVCT2 protein levels in breast cancer cells were predictive of ascorbate uptake and cellular sensitivity to ascorbate cytotoxicity, and this was confirmed *via* overexpression and gene knockdown *in vitro* (69). Furthermore, *in vivo* tumor response to ascorbate administration (1 g/kg/day) correlated with increased SVCT2 protein levels in xenografts (69), although tumor levels of ascorbate were not assessed. Western blot (*n* = 20) and immunohistochemistry (*n* = 92) in breast cancer patients indicated an inverse relationship between SVCT2 protein levels in tumor tissue and hormone receptor status, with low SVCT2 levels in normal tissue (69), again without ascorbate measurements.

The monoclonal antibody cetuximab, used for the treatment of several types of metastatic cancer, inhibits the human epidermal growth factor receptor, and KRAS-mutant tumors are resistant to cetuximab (70). SVCT2 expression sensitized KRAS-mutant human colon cancer cells to combined administration of ascorbate and cetuximab *in vitro* (27). Combination treatment also reduced tumor growth in KRAS-mutant xenograft tumors dependent on SVCT2 levels; intracellular or tumor ascorbate levels have again not been measured (27). In a recent study, ascorbate was also shown to act synergistically with the multikinase inhibitor sorafenib through dysregulation of calcium homeostasis, in addition to H_2_O_2_ production, in a hepatocellular carcinoma cell line (71). Elucidating the role of vitamin C transporters in this setting might further clarify the mechanism of action of ascorbate in modulating the cytotoxicity of chemotherapeutics.

## Concluding Remarks and Future Directions

Ascorbate is critical for many enzymatic reactions in the body and needs to be sufficiently taken up from the diet and distributed into different body compartments. Blood ascorbate levels in cancer patients are measured infrequently, and data are inconsistent in different studies. Importantly, tumor tissue levels in cancer patients have only been analyzed in two studies to date (17, 18). How ascorbate transport proteins are regulated at both the transcriptional and posttranslational level is still not fully understood. There are only three studies that report SVCT protein status in human cancer tissue and none of these report associated ascorbate levels (49, 68, 69). Further research is needed to evaluate if expression and localization of ascorbate transporters in tumors could predict ascorbate uptake, and thus serve as biomarkers for potential therapeutic effect in patients undergoing high dose ascorbate infusion.

## Author Contributions

GD conceived the study; CW collected data from the literature and composed the review; and CW, EP, and GD edited, refined, and finalized the manuscript.

## Conflict of Interest Statement

The authors declare that the research was conducted in the absence of any commercial or financial relationships that could be construed as a potential conflict of interest.

## Abbreviations

5-FU, 5-fluorouracil; CLL, chronic lymphocytic leukemia; SLC23, solute carrier gene family 23; SLC2, solute carrier gene family 2; SVCT, sodium-dependent vitamin C transporter; DHA, dehydroascorbate; GLUTs, glucose transporters; Gulo, l-gulonolactone oxidase; SNP, single nucleotide polymorphism.
